# Inconsistent standard of care for tuberculosis screening and preventive therapy before initiating systemic psoriasis treatment

**DOI:** 10.1111/ddg.15948

**Published:** 2025-11-28

**Authors:** Christoph Zeyen, Ruben Heuer, Brit Haecker, Tom Schaberg, Alexander Nast

**Affiliations:** ^1^ Department of Dermatology Venereology and Allergology Division of Evidence‐Based Medicine (dEBM) Charité – Universitätsmedizin Berlin corporate member of Freie Universität Berlin and Humboldt‐Universität zu Berlin Berlin Germany; ^2^ German Central Committee against Tuberculosis (DZK) Berlin Germany

**Keywords:** IL‐17 antibodies, IL‐23 antibodies, preventive tuberculosis therapy, psoriasis, tuberculosis

## Abstract

**Background and Objectives:**

Preventive tuberculosis (TB) therapy before initiating MTX or IL‐17/IL‐23/IL‐12/23p40 inhibitors for latent tuberculosis infection (LTBI) is supported by indirect evidence of TB reactivations with TNF inhibitors. However, direct evidence for MTX or IL‐17/IL‐23/IL‐12/23p40 inhibitors is limited. To better evaluate the risk of TB reactivation, data on LTBI patients exposed to these medications without preventive TB therapy are necessary. This study was conducted as part of the update of the European and German psoriasis guidelines aimed to assess current LTBI screening and preventive treatment practices.

**Patients and Methods:**

An online survey was distributed via German, European and international dermatological societies, yielding 326 complete responses.

**Results:**

LTBI screening was performed by 45% of respondents before MTX initiation and 95% before IL‐17/IL‐23/IL‐12/23p40 inhibitors. Preventive TB therapy was initiated “always” or “almost always” in 38% of MTX cases and “never” or “almost never” in 31%. For IL‐17/IL‐23/IL‐12/23p40 inhibitors, preventive TB therapy was used in 66% of cases “always” or “almost always,” 16% case‐by‐case and 9% “never” or “almost never.”

**Conclusions:**

LTBI screening and preventive TB therapy for MTX lack standardization. While screening is common for IL‐17/IL‐23/IL‐12/23p40 inhibitors, a significant proportion of LTBI patients receive these treatments without preventive TB therapy.

## INTRODUCTION

There is evidence indicating an increased risk of reactivation of latent tuberculosis infection (LTBI) during the use of tumor necrosis factor inhibitors (TNFi).^1^, ^2^ A significant number of active tuberculosis (TB) cases have been documented and reported.^3^, ^4^ Consequently, major guidelines recommend LTBI screening and preventive TB therapy (PT) prior to initiating TNFi.^5^, ^6^, ^7^ On the other hand, for conventional psoriasis therapies as acitretin, ciclosporin, fumarates as well as for the small molecule apremilast, guidelines do not raise TB concerns and do not recommend an LTBI screening prior to the initiation of these agents.^5^

When administering more modern biologics, such as IL‐17/IL‐23/IL‐12/23p40 inhibitors or the small molecule deucravacitinib, most guidelines follow the approach used for TNFi and also advocate for screening and PT in LTBI patients.^5^, ^6^ This rationale is primarily based on indirect evidence, as reported cases of TB reactivations under these medications are rare,^8^ and randomized clinical trials with this drugs on reactivation of TB are lacking.^9^ As it stands, meta‐analyses with observational data do not indicate a significant risk of reactivation.^10^, ^11^ Austrian societies already advocate another approach in a consensus statement and advise against PT for LTBI patients receiving IL‐17 inhibitors because they consider the TB risk with these agents to be low.^12^ A panel of international psoriasis experts from the *Skin Inflammation and Psoriasis International Network* (SPIN) advocates for an even more flexible approach to PT in patients with LTBI. They recommend that preventive therapy be waived when initiating IL‐17 or IL‐23 inhibitors, as well as apremilast, if the risk of toxicity or drug‐drug interactions is high.^11^ Additionally, they suggest that PT may also be omitted when starting these treatments, even if the risks of PT toxicity or drug‐drug interactions are minimal.^11^

For methotrexate (MTX), available guidelines often lack any clear directives on how to manage LTBI in patients when initiating this commonly prescribed psoriasis treatment. Given that IL‐17/IL‐23/IL‐12/23p40 inhibitors as well as MTX are used to treat psoriasis, rheumatoid arthritis and chronic inflammatory bowel diseases, this issue is relevant not only to dermatologists but also to rheumatologists and gastroenterologists.

In most cases, LTBI screening is performed using interferon‐gamma release assays (IGRA), often in combination with a chest X‐ray to exclude active TB disease.^13^ While these diagnostics are crucial for assessing the patient's individual risk of TB reactivation, they also represent a significant cost to healthcare systems and a potential delay in start of systemic therapy.^14^, ^15^ If the risk of reactivation is determined to be very low for particular systemic agents, these expenses could potentially be reduced, allowing for more efficient allocation of resources. Additionally, this could accelerate the start of systemic psoriasis therapies for practitioners without immediate access to an x‐ray because they would not have to refer their patients to a radiologist first.

The *World Health Organization* and the German national TB guidelines recommend, if indicated, PT with one of the following regimens: a 3‐month course of isoniazid plus rifampicin, 4 months of rifampicin, or a 6‐ to 9‐month course of isoniazid.^16^, ^17^ Initiating biologic treatment is considered safe after 4 to 8 weeks of PT, although this recommendation is not evidence‐based. Such delays can hinder timely disease control and negatively impact patient outcomes, particularly in individuals with high disease burden or limited therapeutic alternatives. Avoiding unnecessary preventive therapy (PT) should be an objective whenever risk assessment permits, given the potential adverse effects of these commonly recommended drugs, including hepatotoxicity, neurotoxicity, and the risk of drug interactions, particularly in patients receiving multiple medications.^18^, ^19^

To obtain direct evidence of the reactivation risk – or the absence thereof – it is essential to examine reported cases of tuberculosis reactivation during the use of MTX and IL‐17/IL‐23/IL‐12/23p40 inhibitors. This should be correlated with cases where patients were exposed to these medications without undergoing PT. The reported cases need to be contextualized in relation to the overall exposed patient population. However, accurately defining and understanding this patient cohort presents a significant challenge.

If dermatologists consistently conduct screening and administer PT, the incidence of reactivation cases may be very low and the reactivation risk underestimated. Conversely, if dermatologists frequently administer MTX and IL‐17/IL‐23/IL‐12/23p40 inhibitors to LTBI patients without PT or fail to screen high‐risk LTBI populations, the low number of reported cases could signify a low reactivation risk.

This survey was conducted as part of European and German guideline development efforts within the framework of updating the S3 guideline on systemic psoriasis treatment. The primary objective was to assess current practices for LTBI screening and the use of preventive tuberculosis therapy before starting systemic psoriasis therapies, specifically MTX and IL‐17/IL‐23/IL‐12/23p40 inhibitors, to estimate the proportion of patients at risk for tuberculosis reactivation. To date, no study has attempted to estimate the proportion of patients in this specific context. Additionally, current practices were surveyed for other therapeutic agents, including acitretin, ciclosporin, fumarates, apremilast and deucravacitinib.

## MATERIAL AND METHODS

### Study Design

This exploratory quantitative cross‐sectional study was conducted using an anonymous online survey. A survey link was distributed to dermatologists via mailing lists from German dermatological societies – *German Society of Dermatology* (DDG; Deutsche Dermatologische Gesellschaft), *Association of German Dermatologists* (BVDD; Berufsverband Deutscher Dermatologen) and *PSONET, German Psoriasis Networks* (Regionale Psoriasisnetze in Deutschland) – as well as from European and international societies, including the *European Dermatology Forum* (EDF), *International Psoriasis Council* (IPC) and *Skin Inflammation and Psoriasis International Network* (SPIN).

The survey was developed in collaboration with senior dermatologists and tuberculosis specialists to ensure methodological rigor and clinical relevance. The survey included multiple‐choice questions, Likert scales and open‐ended questions to gather data on dermatologists' practices and attitudes toward LTBI screening and PT.

The survey included questions about LTBI screening practices before starting systemic antipsoriatic therapies, specifically MTX, IL‐17, IL‐23 and IL‐12/23p40 inhibitors, as well as acitretin, ciclosporin, fumarates, apremilast and deucravacitinib. Respondents were also asked whether they initiate PT in cases of LTBI before administering each of these antipsoriatic therapies.

All dermatologists with access to the societies’ mailing lists were eligible to participate. The only inclusion criterion was active involvement in the treatment of patients with psoriasis. Demographic information about the respondents, such as form of institution and psoriasis specific experience have been collected to understand the sample characteristics.

Ethical considerations, such as ensuring data confidentiality were adhered to throughout the study.

### Data Collection

Data collection occurred over a period of 2 months, starting in November 2023, with at least one reminder sent approximately 4 weeks after the initial mailing. Responses were recorded anonymously. LimeSurvey Community Edition Version 6.2.1+230807 was employed for both the design and implementation of the survey.

### Data Analysis

Data analysis involved summarizing survey responses, calculating proportions and identifying trends or patterns in dermatologists' practices. Descriptive statistics were used to determine the frequency and distribution of LTBI screening practices, as well as the proportion of dermatologists administering PT prior to initiating systemic antipsoriatic therapies.

Statistical analyses were performed with R version number 4.3.1. (http://www.r‐project.org).

### Ethics

The survey was approved by the local ethics committee at Charité Berlin (application number EA4/208/23).

## RESULTS

A total of approximately 8,785 dermatologists were contacted through email distributions across various mailing lists. Of these, 7,165 (81.56%) experts were reached through German channels, while 1,620 (18.44%) were contacted via international ones.

A total of 326 dermatologists provided complete responses to the survey, yielding a response rate of 3.71%. Of these respondents, 147 (45.09%) were contacted via German mailing lists, 179 (54.91%) via international ones. For the individual response rates and all following results per mailing list see online appendix 1–4.

All respondents were clinically active practitioners. 309 (94.79%) dermatologists were board‐certified specialists, while 17 (5.21%) were medical doctors currently in specialist training. Practitioners were asked to self‐rate their expertise in psoriasis treatment on a scale from 0 (no expertise) to 10 (maximum expertise). A total of 92.02% of respondents rated their expertise at 7 or higher, indicating a self‐perceived proficiency level within the upper third of the provided scale.

Of the dermatologists surveyed, 146 (44.79%) reported working in a medical practice, while 174 (53.37%) indicated employment in a clinical setting. An additional 6 respondents (1.84%) reported working in settings other than these two.

### Tuberculosis Screening Measures

Across all responders, 50.61% reported conducting both IGRA and chest x‐ray for screening purposes, while 35.28% solely rely on IGRA. Additionally, 14.11% reported utilizing “other” screening measures, which were not further specified. It is likely that most of these cases involved the use of a tuberculin skin test.

### MTX, TNFi and IL‐17/IL‐23/IL‐12/23p40 inhibitors – Screening for LTBI

MTX, TNFi and IL‐17/IL‐23/IL‐12/23p40 inhibitors were identified as primary focus therapies by the European and German psoriasis guideline groups due to an assumed controversy among experts. Results for these therapies are presented separately from those for conventional treatments and small molecules.

**FIGURE 1:**
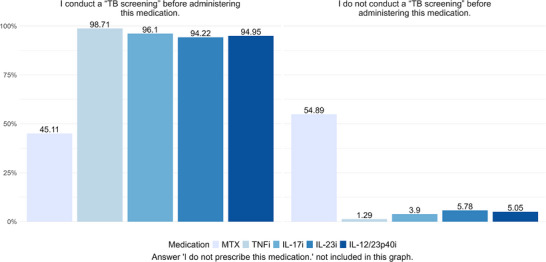
Screening for LTBI prior to administering psoriasis therapy – answers to the question “Before commencing treatment with which of the following medications do you typically perform a tuberculosis screening?”.

Participants were asked, “Before commencing treatment with which of the following medications do you typically perform tuberculosis screening?”. Prior to initiating immunomodulatory psoriasis therapy with MTX, 45.11% of dermatologists perform LTBI screening before treatment initiation, whereas 54.89% do not screen for LTBI in these cases. Before initiating TNF inhibitors, 98.71% of respondents reported conducting LTBI screening, while 1.29% did not. Before starting a therapy with IL‐17, IL‐23, or IL‐12/23p40 inhibitors, LTBI screening is conducted in the majority of cases (96.1% for IL‐17 inhibitors, 94.22% for IL‐23 inhibitors and 94.95% for IL‐12/23p40 inhibitors). However, in a notable proportion of cases, no screening is performed (Figure 1).

### MTX, TNFi and IL‐17/IL‐23/IL‐12/23p40 inhibitors – Preventive therapy in case of LTBI

Experts were asked: “You are presented with a patient who has as latent tuberculosis infection (positive IGRA testing) and a normal chest X‐ray. Active tuberculosis has been ruled out. There is an indication for initiating systemic antipsoriatic therapy. Additionally, there are no other risk factors that would increase the likelihood of tuberculosis reactivation. For which of the following treatments would you administer preventive tuberculosis therapy?”

For MTX, PT is used in 37.54% of LTBI cases, with respondents reporting “always” or “almost always.” However, in 31.23% of cases, PT is “never” or “almost never” performed before beginning MTX, while 21.14% of respondents choose to initiate PT selectively, based on shared decision‐making with the patient.

PT is implemented in 79.55% of LTBI cases when prescribing TNF inhibitors, with respondents indicating “always” or “almost always.” Conversely, 5.11% reported that they “never” or “almost never” initiate PT before starting TNF inhibitors and 5.75% opted for PT “in some cases” following shared decision‐making with the patient.

**FIGURE 2:**
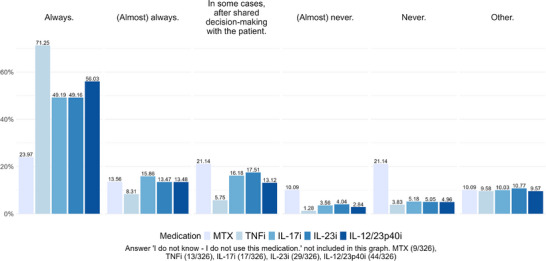
Preventive tuberculosis therapy prior to administering psoriasis therapy – answers to the question “For which of the following treatments would you administer preventive tuberculosis therapy such as rifampicin (for 4 months), isoniazid + rifampicin (for 3 months) or isoniazid (for 9 months)?”.

The distribution of responses regarding the initiation of PT before IL‐17, IL‐23 and IL‐12/23p40 inhibitors is illustrated in Figure 2. Overall, PT is administered in approximately two‐thirds of cases (“always” or “almost always”) across all these inhibitors. Interestingly, PT is “never” or “almost never” provided in 8.74% of cases for IL‐17 inhibitors, 9.09% for IL‐23 inhibitors and 7.8% for IL‐12/23p40 inhibitors.

### Conventional systemic agents and small molecules

While MTX, TNFi, IL‐17, IL‐23 and IL‐12/23p40 inhibitors were the focus therapeutics for the guideline groups, the current standard of care for conventional therapies and newly approved small molecules was also assessed. These additional therapies were initially expected to generate less controversy among experts. For conventional agents, including acitretin, ciclosporin and fumarates, as well as the small molecule apremilast, guidelines and summaries of product characteristics (SmPC) do not recommend TB prevention measures.^5^, ^20^ In contrast, LTBI screening and PT are advised for the recently approved small molecule deucravacitinib (2023), partly due to the limited real‐world data available. Survey results for these agents are presented separately in this manuscript, with an overview provided in Table 1 and 2.

**TABLE 1:** LTBI screening prior to conventional agents and small molecules.

LTBI screening prior to …	LTBI screening n (%)	No LTBI screening n (%)	I do not prescribe this medication n (%)
Acitretin	13 (3.99)	279 (85.58)	34 (10.43)
Ciclosporin	95 (29.14)	183 (56.13)	48 (14.72)
Fumarates	17 (5.21)	191 (58.59)	118 (36.2)
Apremilast (PDE‐4 inhibitor)	79 (24.23)	162 (49.69)	85 (26.07)
Deucravacitinib (TYK2 inhibitor)	154 (47.24)	15 (4.6)	157 (48.16)

**TABLE 2:** Preventive tuberculosis therapy prior to conventional agents and small molecules.

Preventive TB therapy prior to …	Always n (%)	(Almost) always n (%)	In some cases, after shared decision‐making with the patient n (%)	(Almost) never n (%)	Never n (%)	Other n (%)
Acitretin	18 (6.1)	1 (0.34)	24 (8.14)	35 (11.86)	195 (66.1)	22 (7.46)
Ciclosporin	58 (21.09)	30 (10.91)	50 (18.18)	30 (10.91)	78 (28.36)	29 (10.55)
Fumarates	15 (7.46)	6 (2.99)	17 (8.46)	21 (10.45)	122 (60.7)	20 (9.95)
Apremilast (PDE‐4 inhibitor)	38 (15.38)	13 (5.26)	33 (13.36)	41 (16.6)	96 (38.87)	26 (10.53)
Deucravacitinib (TYK2 inhibitor)	85 (49.71)	23 (13.45)	18 (10.53)	6 (3.51)	14 (8.19)	25 (14.62)

## DISCUSSION

The survey reveals substantial variability in the practices regarding LTBI screening and preventive tuberculosis therapy at the initiation of antipsoriatic therapy. Notably, only about half of the practitioners perform both IGRA and chest X‐rays for screening while the other half relies on IGRA only. This discrepancy may reflect the logistical challenges associated with referring patients for chest X‐rays, which can introduce significant delays in starting treatment. It is important to note that IGRA results can yield false negative results, particularly in patients who are already on immunosuppressive therapy.^21^ Therefore, when testing for LTBI is indicated, it should ideally be performed before the initiation of immunosuppressive therapy (particularly TNFi) to reduce the risk of false‐negative results. It has been reported that ELISpot may have a slightly higher sensitivity than QuantiFERON in patients with active TB and indeterminate results.^22^ Current tuberculosis guidelines identify specific groups at elevated risk for TB, recommending the integration of X‐ray screening alongside IGRA in these cases.^7^, ^17^

The results concerning LTBI screening before antipsoriatic therapy are particularly interesting for MTX, with nearly half of patients being screened and the other half not. The high rate of screening prior to the initiation of TNF inhibitors is unsurprising, while the somewhat lower screening rates for IL‐17, IL‐23 and IL‐12/23p40 inhibitors may reflect the estimated lower risk of LTBI reactivation with these therapies.

The results for PT closely mirror the screening frequencies, with approximately one‐third of patients receiving PT in cases of LTBI prior to MTX initiation. PT rates before starting TNF inhibitors are significantly higher, aligning with the widely accepted understanding of the substantial reactivation risk associated with these drugs. Similar to the screening rates, PT rates for IL‐17, IL‐23 and IL‐12/23p40 inhibitors are lower, possibly indicating a reduced concern about reactivation with these therapies.

In clinical practice, many biologic‐naive patients are initially treated with biosimilar adalimumab due to economic incentives or insurance‐related restrictions. In these cases, LTBI screening is mandatory upfront, irrespective of possible treatment changes later on. This may support a pragmatic, sequential approach in countries facing such constraints: performing LTBI screening before initiating systemic therapy, followed by adalimumab treatment if the result is negative or once PT has been initiated. In cases where PT is contraindicated, switching to IL‐17 or IL‐23 inhibitors without PT may be considered. This strategy could be further explored in future guideline updates. In contrast, in countries where IL‐17, IL‐23 or IL‐12/23p40 inhibitors are commonly used as first‐line systemic agents, alternative approaches may apply.

In patients with a history of LTBI who have already completed preventive therapy or treatment for active TB, reactivation risk is markedly reduced and repeated PT may not be required. However, when feasible, alternatives to TNFis may be considered to further minimize TB risk.

A further noteworthy finding is the high variability in patient management for conventional therapies, that were initially considered non‐controversial, due to clear guideline and SmPC recommendations. Despite guidelines advising against it and no data for an increased TB reactivation risk is available, nearly 30% of practitioners conduct TB screening before initiating ciclosporin and around 24% do so before starting apremilast. Even more concerning is that over 30% of dermatologists prescribe potentially harmful tuberculosis medication before ciclosporin and over 20% before apremilast.

Going back to the initial attempt to estimate rates of patients with LTBI that are exposed to MTX, TNFi or IL‐17/IL‐23/IL‐12/23p40 inhibitors without PT, multiple conclusions can be drawn.

Fewer than 50% of practitioners conduct any screening prior to the initiation of MTX. Moreover, less than 40% initiate PT “always” or “almost always” in cases of positive LTBI screening. Consequently, a significant number of patients with LTBI are likely receiving MTX without PT.

The rates of practitioners initiating IL‐17, IL‐23, or IL‐12/23p40 inhibitors without screening and/or without PT is not high, but with about 3%–5% percent of the practitioners not performing a screening and 6%–9% not initiating PT, the number of patients exposed is relevant and some cases of TB reactivations could be expected.

Considering the epidemiology, prevalence and incidence of TB and LTBI, along with the thousands of patient‐years of exposure to specific biologics, one would anticipate observing a corresponding number of reactivation cases if a significant reactivation risk existed. LTBI is estimated to affect 20%–25% of the global population,^23^, ^24^ with a prevalence of 11%–14% in Europe.^23^, ^24^ Without immunomodulating therapy, the lifetime risk of reactivation for people with untreated LTBI is considered to be 5%–10%.^25^, ^26^, ^27^ When immunomodulating therapy is administered, reactivations are most likely to occur within the first 6 months of treatment,^28^ making them detectable relatively quickly. Given these established rates, any notable increase in reactivation cases would likely be reflected in clinical data or reported case reports.

The fact that nearly all respondents were board‐certified dermatologists, with over 90% demonstrating psoriasis‐specific expertise rated at ≥ 7/10, further validates the findings. The decision by dermatologists not to implement screening or PT appears to be intentional, guided by clinical experience rather than a lack of knowledge.

### Limitations

Since the data collection was performed anonymously, it cannot be guaranteed that individuals provided their answers only once. The survey did not capture data on specific patient demographics, disease severity or comorbidity or additional risk factors which could influence practices related to LTBI screening and PT. The response rate of approximately 4% may introduce non‐response bias and limit the generalizability of the results. It is conceivable that dermatologists with a particular interest in psoriasis were more likely to participate than those with a broader, non‐specialized dermatological focus. Nevertheless, the high level of psoriasis‐specific expertise among respondents lends credibility to the findings. Although the use of specific mailing lists may have excluded some dermatologists, the balanced distribution of respondents between specialist practices (approximately half) and clinical settings underscores the robustness of the results across the full spectrum of dermatological care.

A further limitation lies in the geographic distribution of respondents, which was primarily centered on Germany, with additional participation from other European and international dermatologists. Although 179 responses were received from non‐German participants, the number was insufficient to allow for meaningful country‐specific stratification. As a result, potential regional differences in tuberculosis incidence, national healthcare policies and clinical practice patterns could not be fully explored. These factors may limit the generalizability of the findings to regions with different epidemiological or healthcare contexts. Additionally, repeated or periodical LTBI testing was not addressed, although it may be relevant for patients receiving long‐term immunosuppressive therapy, particularly in countries with a high incidence of tuberculosis. The number of patients treated daily per respondent may vary.

Overall, subtle but always continuously reproduced differences between TNFi and the other biologics confirm clear understanding/data quality.

## CONCLUSIONS

There is considerable heterogeneity among dermatologists regarding LTBI screening and PT prior to initiating systemic psoriasis treatments. Notably, there is variability in screening approaches, with some practitioners using IGRA alone while others combine IGRA with chest X‐rays.

While LTBI screening is routinely performed before initiating IL‐17, IL‐23 and IL‐12/23p40 inhibitors, it is less consistently applied prior to MTX. PT is commonly initiated before starting IL‐17, IL‐23 and IL‐12/23p40 inhibitors. However, a subset of dermatologists adopts an individualized approach to screening and PT with these therapies, leading to a proportion of patients being exposed to IL‐17, IL‐23 or IL‐12/23p40 inhibitors without PT.

The findings presented in this study underscore the need for refining dermatological guidelines at both German and European levels, potentially promoting a more standardized approach. The SPIN position paper already provides a consensus‐based framework for treating LTBI patients with IL‐17 and IL‐23 inhibitors without PT. Future research should focus on patients with LTBI exposed to the medications where the reactivation risk remains unclear, ideally in form of a multinational registry. Multidisciplinary guidelines for LTBI screening and PT should be regularly updated in response to emerging evidence. Balancing benefits and harms of preventive tuberculosis medication is desirable, while ensuring cost efficiency and practicality for healthcare systems.

## CONFLICT OF INTEREST STATEMENT

None.

## Supporting information



Supplementary information
