# Supplementary material for: Inconsistent standard of care for tuberculosis screening and preventive therapy before initiating systemic psoriasis treatment
Source: J Dtsch Dermatol Ges. 2025 Nov 28;24(6):737–44. doi: 10.1111/ddg.15948 (PMC13238368; doi:10.1111/ddg.15948)
Supplement: Supplementary file 1 — Supplementary information [file DDG-24-737-s001.docx]

# Appendix

**Appendix 1:** Response Rates by Mailing List

|  | **All Mailing Lists n (%)** | **BVDD n (%)** | **DDG* n (%)** | **PsoNet n (%)** | **EDF n (%)** | **IPC n (%)** | **SPIN n (%)** |
| --- | --- | --- | --- | --- | --- | --- | --- |
| Number of Recipients | **8785 (100)** | 3421 (100) | 3635 (100) | 109 (100) | 253 (100) | 1217 (100) | 150 (100) |
| Number of complete Responses | **326 (3.71)** | 122 (3.57) | 0 (0) | 25 (22.94) | 23 (9.09) | 94 (7.72) | 62 (41.33) |
| *Via DDG mailing list no responses received. The survey, which was behind a login wall, was not accessed. | | | | | | | |

**Appendix 2:** LTBI Screening Measures by Mailing List

| **Screening Measures** | **All Mailing Lists n (%)** | **BVDD n (%)** | **DDG* n (%)** | **PsoNet n (%)** | **EDF n (%)** | **IPC n (%)** | **SPIN n (%)** |
| --- | --- | --- | --- | --- | --- | --- | --- |
| IGRA + Chest X-ray | **165 (50.61)** | 69 (56.56) | 0 (0) | 17 (68) | 9 (39.13) | 41 (43.62) | 29 (46.77) |
| Interferon-Gamma Release Assay (IGRA), e.g., Quantiferon test | **115 (35.28)** | 52 (42.62) | 0 (0) | 7 (28) | 11 (47.83) | 26 (27.66) | 19 (30.65) |
| Other | **46 (14.11)** | 1 (0.82) | 0 (0) | 1 (4) | 3 (13.04) | 27 (28.72) | 14 (22.58) |
| **Total** | **326 (100)** | **122 (100)** | **0 (100)** | **25 (100)** | **23 (100)** | **94 (100)** | **62 (100)** |
| *Via DDG mailing list no responses received. The survey, which was behind a login wall, was not accessed. | | | | | | | |

**Appendix 3:** LTBI Screening Prior to Initiation of Psoriasis Therapy by Mailing List

| **“Before commencing treatment with which of the following medications, do you typically perform a tuberculosis screening?”** | | **All Mailing Lists** | | **BVDD** | | **DDG*** | | **PsoNet** | | **EDF** | | **IPC** | | **SPIN** | |
| --- | --- | --- | --- | --- | --- | --- | --- | --- | --- | --- | --- | --- | --- | --- | --- |
|  |  | **Yes n(%)** | **No n(%)** | **Yes n(%)** | **No n(%)** | **Yes n(%)** | **No n(%)** | **Yes n(%)** | **No n(%)** | **Yes n(%)** | **No n(%)** | **Yes n(%)** | **No n(%)** | **Yes n(%)** | **No n(%)** |
| Conventionals | Acitretin | **13 (4.45)** | **279 (95.55)** | 5  (4.76) | 100 (95.24) | 0 (0) | 0 (0) | 0  (0) | 23  (100) | 1 (4.55) | 21 (95.45) | 3 (3.57) | 81 (96.43) | 4  (6.9) | 54 (93.1) |
|  | Ciclosporin | **95 (34.17)** | **183 (65.83)** | 20 (22.73) | 68 (77.27) | 0 (0) | 0 (0) | 6  (27.27) | 16  (72.73) | 5 (22.73) | 17 (77.27) | 32 (36.78) | 55 (63.22) | 32 (54.24) | 27 (45.76) |
|  | Fumarates | **17 (8.17)** | **191 (91.83)** | 9  (7.5) | 111 (92.5) | 0 (0) | 0 (0) | 0  (0) | 25 (100) | 0  (0) | 15 (100) | 2 (9.09) | 20 (90.91) | 6 (23.08) | 20 (76.92) |
|  | Methotrexate | **143 (45.11)** | **174 (54.89)** | 63 (52.94) | 56 (47.06) | 0 (0) | 0 (0) | 15  (60) | 10  (40) | 4 (18.18) | 18 (81.82) | 34 (37.36) | 57 (62.64) | 27  (45) | 33 (55) |
| Biologicals | TNF inhibitors | **305 (98.71)** | **4 (1.29)** | 118 (99.16) | 1 (0.84) | 0 (0) | 0 (0) | 25 (100) | 0  (0) | 22 (100) | 0  (0) | 82 (98.80) | 1  (1.2) | 58 (96.67) | 2 (3.33) |
|  | IL 17 inhibitors | **296 (96.1)** | **12 (3.9)** | 119 (98.35) | 2 (1.65) | 0 (0) | 0 (0) | 24  (96) | 1  (4) | 20 (90.91) | 2  (9.09) | 77 (95.06) | 4 (4.94) | 56 (94.92) | 3 (5.08) |
|  | IL 23 inhibitors | **277 (94.22)** | **17 (5.78)** | 118 (97.52) | 3 (2.48) | 0 (0) | 0 (0) | 24  (96) | 1  (4) | 18 (85.71) | 3 (14.29) | 68 (93.15) | 5 (6.85) | 49 (90.74) | 5 (9.26) |
|  | Ustekinumab  (IL-12/23p40) | **263 (94.95)** | **14 (5.05)** | 106 (98.15) | 2 (1.85) | 0 (0) | 0 (0) | 25 (100) | 0  (0) | 18 (85.71) | 3 (14.29) | 65 (94.20) | 4 (5.80) | 49 (90.74) | 5 (9.26) |
| Small Molecules | Apremilast  (Phosphodiesterase-4 inhibitor) | **79 (32.78)** | **162 (67.22)** | 39 (37.14) | 66 (62.86) | 0 (0) | 0 (0) | 4  (18.18) | 18  (81.82) | 1 (6.25) | 15 (93.75) | 20 (36.36) | 35 (63.64) | 15 (34.88) | 28 (65.12) |
|  | Deucravacitinib  (TYK2 inhibitor) | **154 (91.12)** | **15 (8.88)** | 77 (93.90) | 5  (6.10) | 0 (0) | 0 (0) | 16  (100) | 0  (0) | 10 (83.33) | 2  (16.67) | 30 (93.75) | 2 (6.25) | 21 (77.78) | 6 (22.22) |
| “I do not prescribe this medication” not included. | | | | | | | | | | | | | | | |
| *Via DDG mailing list no responses received. The survey, which was behind a login wall, was not accessed. | | | | | | | | | | | | | | | |

**Appendix 4:** Preventive TB Therapy in Case of LTBI Prior to Initiation of Psoriasis Therapy by Mailing List

|  | **Preventive TB therapy prior to administering therapy – Answers to question “For which of the following treatments would you administer preventive tuberculosis therapy, such as Rifampicin (for 4 months), Isoniazid + Rifampicin (for 3 months) or Isoniazid (for 9 months)?”** | | **All Mailing Lists n (%)** | **BVDD n (%)** | **DDG^*^ n (%)** | **PsoNet n (%)** | **EDF n (%)** | **IPC n (%)** | **SPIN n (%)** |
| --- | --- | --- | --- | --- | --- | --- | --- | --- | --- |
| Conventionals | Acitretin | Never. | **195 (66.1)** | 67 (66.34) | 0 (0) | 18 (75) | 15 (71.43) | 57 (64.04) | 38 (63.33) |
|  |  | (Almost) never. | **35 (11.86)** | 8 (7.92) | 0 (0) | 4 (16.67) | 2 (9.52) | 13 (14.61) | 8 (13.33) |
|  |  | In some cases, after shared decision-making with the patient. | **24 (8.14)** | 7 (6.93) | 0 (0) | 1 (4.17) | 3 (14.29) | 8 (8.99) | 5 (8.33) |
|  |  | (Almost) always. | **1 (0.34)** | 0 (0) | 0 (0) | 0 (0) | 0 (0) | 1 (1.12) | 0 (0) |
|  |  | Always. | **18 (6.1)** | 3 (2.97) | 0 (0) | 1 (4.17) | 1 (4.76) | 8 (8.99) | 5 (8.33) |
|  |  | Other | **22 (7.46)** | 16 (15.84) | 0 (0) | 0 (0) | 0 (0) | 2 (2.25) | 4 (6.67) |
|  | Ciclosporin | Never. | **78 (28.36)** | 35 (40.7) | 0 (0) | 6 (30) | 9 (45) | 17 (19.1) | 11 (18.33) |
|  |  | (Almost) never. | **30 (10.91)** | 8 (9.3) | 0 (0) | 3 (15) | 1 (5) | 13 (14.61) | 5 (8.33) |
|  |  | In some cases, after shared decision-making with the patient. | **50 (18.18)** | 9 (10.47) | 0 (0) | 4 (20) | 4 (20) | 20 (22.47) | 13 (21.67) |
|  |  | (Almost) always. | **30 (10.91)** | 7 (8.14) | 0 (0) | 1 (5) | 2 (10) | 12 (13.48) | 8 (13.33) |
|  |  | Always. | **58 (21.09)** | 10 (11.63) | 0 (0) | 3 (15) | 4 (20) | 22 (24.72) | 19 (31.67) |
|  |  | Other | **29 (10.55)** | 17 (19.77) | 0 (0) | 3 (15) | 0 (0) | 5 (5.62) | 4 (6.67) |
|  | Fumarates | Never. | **122 (60.7)** | 74 (62.18) | 0 (0) | 17 (68) | 9 (75) | 9 (39.13) | 13 (59.09) |
|  |  | (Almost) never. | **21 (10.45)** | 11 (9.24) | 0 (0) | 4 (16) | 0 (0) | 6 (26.09) | 0 (0) |
|  |  | In some cases, after shared decision-making with the patient. | **17 (8.46)** | 8 (6.72) | 0 (0) | 2 (8) | 2 (16.67) | 2 (8.7) | 3 (13.64) |
|  |  | (Almost) always. | **6 (2.99)** | 4 (3.36) | 0 (0) | 1 (4) | 0 (0) | 0 (0) | 1 (4.55) |
|  |  | Always. | **15 (7.46)** | 5 (4.2) | 0 (0) | 1 (4) | 1 (8.33) | 5 (21.74) | 3 (13.64) |
|  |  | Other | **20 (9.95)** | 17 (14.29) | 0 (0) | 0 (0) | 0 (0) | 1 (4.35) | 2 (9.09) |
|  | MTX | Never. | **67 (21.14)** | 27 (23.08) | 0 (0) | 4 (16) | 9 (42.86) | 16 (17.39) | 11 (17.74) |
|  |  | (Almost) never. | **32 (10.09)** | 12 (10.26) | 0 (0) | 1 (4) | 1 (4.76) | 11 (11.96) | 7 (11.29) |
|  |  | In some cases, after shared decision-making with the patient. | **67 (21.14)** | 16 (13.68) | 0 (0) | 7 (28) | 6 (28.57) | 21 (22.83) | 17 (27.42) |
|  |  | (Almost) always. | **43 (13.56)** | 11 (9.4) | 0 (0) | 4 (16) | 3 (14.29) | 18 (19.57) | 7 (11.29) |
|  |  | Always. | **76 (23.97)** | 28 (23.93) | 0 (0) | 7 (28) | 2 (9.52) | 23 (25) | 16 (25.81) |
|  |  | Other | **32 (10.09)** | 23 (19.66) | 0 (0) | 2 (8) | 0 (0) | 3 (3.26) | 4 (6.45) |
| Biologicals | TNF inhibitors | Never. | **12 (3.83)** | 8 (6.72) | 0 (0) | 0 (0) | 0 (0) | 4 (4.65) | 0 (0) |
|  |  | (Almost) never. | **4 (1.28)** | 3 (2.52) | 0 (0) | 0 (0) | 0 (0) | 0 (0) | 1 (1.61) |
|  |  | In some cases, after shared decision-making with the patient. | **18 (5.75)** | 9 (7.56) | 0 (0) | 1 (4) | 3 (14.29) | 3 (3.49) | 2 (3.23) |
|  |  | (Almost) always. | **26 (8.31)** | 10 (8.4) | 0 (0) | 3 (12) | 2 (9.52) | 7 (8.14) | 4 (6.45) |
|  |  | Always. | **223 (71.25)** | 65 (54.62) | 0 (0) | 19 (76) | 16 (76.19) | 70 (81.4) | 53 (85.48) |
|  |  | Other | **30 (9.58)** | 24 (20.17) | 0 (0) | 2 (8) | 0 (0) | 2 (2.33) | 2 (3.23) |
|  | IL 17 inhibitors | Never. | **16 (5.18)** | 9 (7.5) | 0 (0) | 1 (4) | 1 (4.76) | 4 (4.88) | 1 (1.64) |
|  |  | (Almost) never. | **11 (3.56)** | 3 (2.5) | 0 (0) | 2 (8) | 0 (0) | 4 (4.88) | 2 (3.28) |
|  |  | In some cases, after shared decision-making with the patient. | **50 (16.18)** | 21 (17.5) | 0 (0) | 3 (12) | 6 (28.57) | 9 (10.98) | 11 (18.03) |
|  |  | (Almost) always. | **49 (15.86)** | 12 (10) | 0 (0) | 5 (20) | 4 (19.05) | 12 (14.63) | 16 (26.23) |
|  |  | Always. | **152 (49.19)** | 52 (43.33) | 0 (0) | 13 (52) | 10 (47.62) | 48 (58.54) | 29 (47.54) |
|  |  | Other | **31 (10.03)** | 23 (19.17) | 0 (0) | 1 (4) | 0 (0) | 5 (6.1) | 2 (3.28) |
|  | IL 23 inhibitors | Never. | **15 (5.05)** | 10 (8.26) | 0 (0) | 1 (4) | 1 (4.76) | 2 (2.7) | 1 (1.79) |
|  |  | (Almost) never. | **12 (4.04)** | 3 (2.48) | 0 (0) | 2 (8) | 0 (0) | 6 (8.11) | 1 (1.79) |
|  |  | In some cases, after shared decision-making with the patient. | **52 (17.51)** | 21 (17.36) | 0 (0) | 2 (8) | 7 (33.33) | 8 (10.81) | 14 (25) |
|  |  | (Almost) always. | **40 (13.47)** | 9 (7.44) | 0 (0) | 7 (28) | 5 (23.81) | 8 (10.81) | 11 (19.64) |
|  |  | Always. | **146 (49.16)** | 55 (45.45) | 0 (0) | 12 (48) | 8 (38.1) | 44 (59.46) | 27 (48.21) |
|  |  | Other | **32 (10.77)** | 23 (19.01) | 0 (0) | 1 (4) | 0 (0) | 6 (8.11) | 2 (3.57) |
|  | Ustekinumab (IL-12/23p40) | Never. | **14 (4.96)** | 9 (8.18) | 0 (0) | 0 (0) | 1 (5) | 4 (5.63) | 0 (0) |
|  |  | (Almost) never. | **8 (2.84)** | 5 (4.55) | 0 (0) | 0 (0) | 0 (0) | 2 (2.82) | 1 (1.79) |
|  |  | In some cases, after shared decision-making with the patient. | **37 (13.12)** | 16 (14.55) | 0 (0) | 1 (4) | 6 (30) | 7 (9.86) | 7 (12.5) |
|  |  | (Almost) always. | **38 (13.48)** | 8 (7.27) | 0 (0) | 9 (36) | 5 (25) | 8 (11.27) | 8 (14.29) |
|  |  | Always. | **158 (56.03)** | 52 (47.27) | 0 (0) | 14 (56) | 8 (40) | 46 (64.79) | 38 (67.86) |
|  |  | Other | **27 (9.57)** | 20 (18.18) | 0 (0) | 1 (4) | 0 (0) | 4 (5.63) | 2 (3.57) |
| Small Molecules | Apremilast | Never. | **96 (38.87)** | 44 (41.12) | 0 (0) | 9 (42.86) | 8 (47.06) | 21 (37.5) | 14 (30.43) |
|  |  | (Almost) never. | **41 (16.6)** | 15 (14.02) | 0 (0) | 4 (19.05) | 2 (11.76) | 8 (14.29) | 12 (26.09) |
|  |  | In some cases, after shared decision-making with the patient. | **33 (13.36)** | 9 (8.41) | 0 (0) | 4 (19.05) | 5 (29.41) | 8 (14.29) | 7 (15.22) |
|  |  | (Almost) always. | **13 (5.26)** | 5 (4.67) | 0 (0) | 1 (4.76) | 1 (5.88) | 3 (5.36) | 3 (6.52) |
|  |  | Always. | **38 (15.38)** | 14 (13.08) | 0 (0) | 1 (4.76) | 1 (5.88) | 14 (25) | 8 (17.39) |
|  |  | Other | **26 (10.53)** | 20 (18.69) | 0 (0) | 2 (9.52) | 0 (0) | 2 (3.57) | 2 (4.35) |
|  | Deucravacitinib | Never. | **14 (8.19)** | 11 (13.25) | 0 (0) | 0 (0) | 1 (11.11) | 2 (5.56) | 0 (0) |
|  |  | (Almost) never. | **6 (3.51)** | 3 (3.61) | 0 (0) | 1 (6.67) | 0 (0) | 0 (0) | 2 (7.14) |
|  |  | In some cases, after shared decision-making with the patient. | **18 (10.53)** | 9 (10.84) | 0 (0) | 1 (6.67) | 2 (22.22) | 3 (8.33) | 3 (10.71) |
|  |  | (Almost) always. | **23 (13.45)** | 6 (7.23) | 0 (0) | 2 (13.33) | 3 (33.33) | 8 (22.22) | 4 (14.29) |
|  |  | Always. | **85 (49.71)** | 35 (42.17) | 0 (0) | 9 (60) | 3 (33.33) | 20 (55.56) | 18 (64.29) |
|  |  | Other | **25 (14.62)** | 19 (22.89) | 0 (0) | 2 (13.33) | 0 (0) | 3 (8.33) | 1 (3.57) |
| “I do not prescribe this medication” not included. | | | | | | | | | |
| *Via DDG mailing list no responses received. The survey, which was behind a login wall, was not accessed. | | | | | | | | | |
